# The Drosophila actin nucleator DAAM is essential for left-right asymmetry

**DOI:** 10.1371/journal.pgen.1008758

**Published:** 2020-04-23

**Authors:** Anil Chougule, François Lapraz, István Földi, Delphine Cerezo, József Mihály, Stéphane Noselli

**Affiliations:** 1 Université Côte D’Azur, CNRS, Inserm, iBV, Nice, France; 2 Biological Research Centre, Hungarian Academy of Sciences, Institute of Genetics, Hungary; Osaka University, JAPAN

## Abstract

Left-Right (LR) asymmetry is essential for organ positioning, shape and function. Myosin 1D (Myo1D) has emerged as an evolutionary conserved chirality determinant in both Drosophila and vertebrates. However, the molecular interplay between Myo1D and the actin cytoskeleton underlying symmetry breaking remains poorly understood. To address this question, we performed a dual genetic screen to identify new cytoskeletal factors involved in LR asymmetry. We identified the conserved actin nucleator DAAM as an essential factor required for both dextral and sinistral development. In the absence of DAAM, organs lose their LR asymmetry, while its overexpression enhances Myo1D-induced *de novo* LR asymmetry. These results show that DAAM is a limiting, LR-specific actin nucleator connecting up Myo1D with a dedicated F-actin network important for symmetry breaking.

## Author summary

Although our body looks symmetrical when viewed from the outside, it is in fact highly asymmetrical when we consider the shape and implantation of organs. For example, our heart is on the left side of the thorax, while the liver is on the right. In addition, our heart is made up of two distinct parts, the right heart and the left heart, which play different roles for blood circulation. These asymmetries, called left-right asymmetries, play a fundamental role in the morphogenesis and function of visceral organs and the brain. Aberrant LR asymmetry in human results in severe anatomical defects leading to embryonic lethality, spontaneous abortion and a number of congenital disorders. Our recent work has identified a particular myosin (Myo1D) as a major player in asymmetry in Drosophila and vertebrates. Myosins are proteins that can interact with the skeleton of cells (called the cytoskeleton) to transport other proteins, contract the cells, allow them to move, etc. In this work, we were able to identify all the genes of the cytoskeleton involved with myosin in left-right asymmetry, in particular a so-called 'nucleator' gene because it is capable of forming new parts of the cytoskeleton necessary for setting up asymmetries.

## Introduction

Left-Right (LR) asymmetry, or chirality, is a universal feature of living organisms. It is essential to organs for their positioning (e.g., heart on the left side), lateralized differentiation (e.g., heart, lungs) and proper directional coiling (e.g., gut, heart tube). The study of LR asymmetry in model organisms has led to the identification of key molecular pathways and symmetry breaking mechanisms [1–3]. While vertebrates use directional movement of cells (chick), ions (Xenopus) or cilia-dependent nodal flow (mouse) as symmetry breaking processes, invertebrates (snail, nematode, Drosophila) establish LR asymmetry mostly through acto-myosin-based mechanisms. In particular, work in Drosophila identified the conserved *myosin1D* (*myo1D*) gene as a major dextral determinant [4,5]. *myo1D* establishes LR asymmetry through interaction with the adherens junction [6,7], Hox genes [8], planar cell polarity [9] and cell death pathways [10]. In flies, several organs are chiral and undergo stereotyped looping in the dextral direction (testis, genitalia, gut)[11,12]. Dextral is the wild type orientation and thus corresponds to the *situs solitus* condition in Drosophila. Loss of *myo1D* function leads to a sinistral or *situs inversus* phenotype, making organs undergo looping in the opposite direction. The existence of two opposite phenotypes and previous genetic data suggest that two pathways exists, one dextral and one sinistral, with dextral being ‘dominant’ over sinistral [8]. To date, the genetic basis of sinistral asymmetry remains uncharacterized in any system, due to the lack of dedicated genetic screens to identify genes with a specific role in sinistral development.

Our recent work showed that *myo1D* is able to induce *de novo* chirality at all biological scales, from molecular to organismal level. Indeed, ectopic expression of *myo1D* in naïve tissues like the larval epidermis or trachea is sufficient to induce their directional twisting [13]. These results indicate that Myo1D is not only necessary for native LR asymmetry but also sufficient to induce *de novo* chirality at multiple scales [13].

Interestingly, recent work showed that *myo1D* is also involved in LR asymmetry of Xenopus and zebrafish [14,15], hence *myo1D* represents a unique dextral determinant whose function is conserved across phyla. These findings, together with the existence of nodal-independent mechanisms for LR development of the heart [16], further suggest that *myo1D*-dependent and actin-based processes may represent a unifying mechanism controlling LR asymmetry in both vertebrates and invertebrates. In further support of this view, recent work identified a mutation in the *diaphanous*1 (*dia1*) gene as being important for controlling dextral chirality of snail shell in *Lymnaea stagnalis* [17–19]. *dia1* belongs to the family of formin genes, encoding conserved factors involved in actin assembly [20,21].

While a role of actin and associated factors emerges as a central mechanism for LR asymmetry establishment across phyla, the exact nature of actin factors and their interplay remain largely unknown. To try addressing these questions, we have undertaken a dedicated genetic screen aiming at identifying novel regulators of dextral and/or sinistral development in Drosophila. In this study, we have screened 539 genes involved in cytoskeleton homeostasis and identified 8 novel candidate genes whose loss-of-function leads to LR asymmetry defects. We have further characterized the role of the formin DAAM (Dishevelled Associated Activator of Morphogenesis), showing that it is a LR-specific actin nucleator essential for *myo1D* function both in native and *de novo* LR asymmetry. Of note, DAAM is also playing a critical role in the sinistral pathway, making it a unique common denominator of Drosophila LR pathways. Our genetic screen further identified *flightless* (*fli*), *chickadee* (*chic*; encoding Profilin) and the src family non-receptor tyrosine kinase *Tec29*, which have previously been identified as regulators of DAAM for actin nucleation and F-actin polymerization [22–24]. Altogether, our results uncover the DAAM pathway as a key regulator providing a specific F-actin network essential for *myo1D*-dependent LR asymmetry.

## Results

### Genetic screen to identify cytoskeleton regulators important for Drosophila LR asymmetry

**Fig 1:**
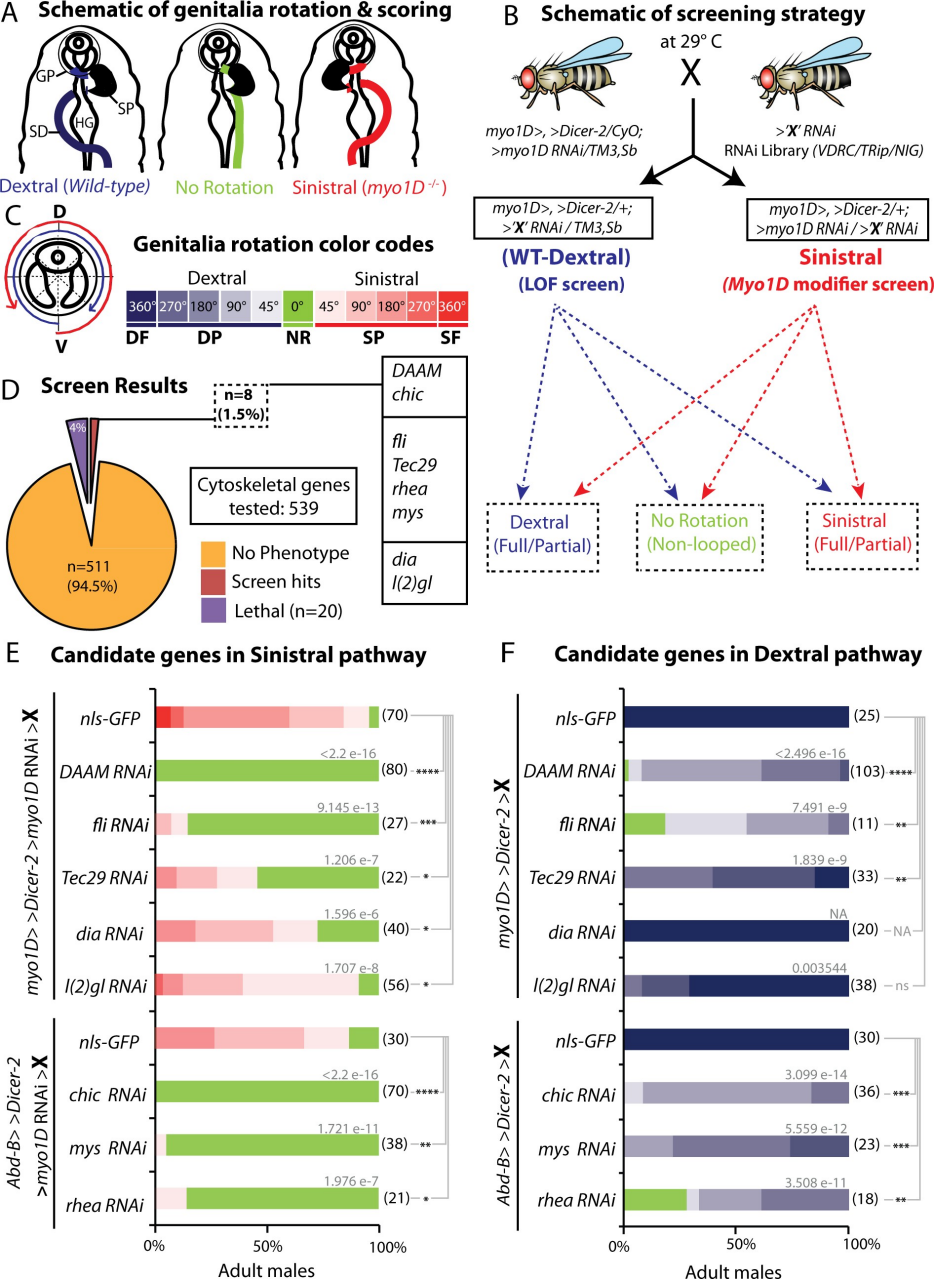
Genetic screen to identify new regulators of LR asymmetry. **A**, Schematic representation of dextral (wildtype, blue spermiduct), sinistral (*myo1D* mutant, red spermiduct) and non-rotated genitalia (green spermiduct). Posterior is up. SD, spermiduct; HG, hindgut; SP, sperm pump; GP, genital plate. **B**, Schematic representation of the dual genetic screen used in this study, allowing screening for potential dextral and/or sinistral factors. Gal4 and UAS are abbreviated as ‘>‘, after or before their name, respectively. **C**, Description of the color code used to describe the extent of genitalia rotation, a schematic genital plate is represented on the left side. D, dorsal; V, ventral; DF, dextral full; DP, dextral partial; NR, no rotation; SP, sinistral partial; SF, sinistral full. **D**, Summary of the screen results. From 539 genes tested, 20 led to lethality and 8 (1.5%) showed a specific LR phenotype (in order of phenotypic strength: *DAAM*, *chic*, *fli*, *Tec29*, *rhea*,*mys*, *dia*, *l(2)gl*)(see E, below). **E**, Phenotype induced by candidate genes in the sinistral pathway, using *myo1D-Gal4* or *Abd-B-Gal4* drivers. Color code as in C. **F**, Phenotype induced by candidate genes in the dextral pathway, using *myo1D-Gal4* or *Abd-B-Gal4* drivers. Color code as in C. In E and F: Numbers in parenthesis on the right side of color bars indicate the number of individuals analyzed for each genotype; bars with different colors represent the percentage of occurrence of a given genitalia posture, as described in C. Numbers above bars indicate Wilcoxon rank sum test p-values. P-value threshold for significance of the difference between compared genotypes is defined as: *: <1e-5; **: <1e-8; ***: <1e-11; ****: <1e-14. NA: not applicable.

Myo1D acts as a dextral determinant, necessary and sufficient to drive dextral development of native LR organs and to induce *de novo* chirality of symmetrical ones [4,5,13]. In the absence of *myo1D* activity, native asymmetrical organs are inverted along the LR axis (mirror image orientation; Fig 1A), hence revealing the existence of a ‘recessive’ sinistral pathway. So far, no gene controlling sinistral development specifically has been identified. In order to get new insights into both dextral and sinistral LR pathways, we have designed a dual genetic screen in which candidate genes with a role in either dextral, sinistral or both pathways could be potentially identified (Fig 1B). A tester line expressing *UAS-myo1D-RNAi* in the *myo1D*-expression domain (*myo1D-gal4*) is crossed to a collection of UAS-lines expressing RNAi against selected genes (*UAS-‘X’-RNAi*). The progeny of this cross leads to i) flies expressing *UAS-‘X’-RNAi* in the *myo1D* domain, allowing to test the role of candidate genes in the dextral pathway, and ii) flies co-expressing *UAS-myo1D-RNAi* (sinistral phenotype) and *UAS-‘X’-RNAi*, allowing to screen for candidate genes involved in the sinistral pathway. Through this screening scheme, we aimed at identifying cytoskeleton regulatory genes involved in LR asymmetry. Based on the Gene List Annotation for Drosophila (GLAD) database [25] and Perkins et al. [26], we have selected 539 candidate genes annotated as playing a potential role in actin or microtubule cytoskeleton regulation (S1 Table), and tested their involvement in dextral and/or sinistral development. 1–3 RNAi lines targeting each individual candidate gene were used for screening. In wildtype males, genitalia rotate by 360° clockwise (or dextral) while rotation is inverted in *myo1D-RNAi* males (sinistral; ranging from -45° to -360°). The extent of genitalia rotation was used as an external marker to determine the modifying effect of candidate gene silencing in either the dextral or the sinistral pathway (Fig 1C). Among 539 candidate genes tested, most (511; 94.5%) did not show any phenotype while 20 (4%) led to lethality (Fig 1D) using either *myo1D-gal4* or an alternative Gal4 line (*Abd-B-gal4*). Interestingly, out of 539 genes, 8 (1.5%) (*chickadee (chic)*, *DAAM*, *lethal(2) giant larvae (l(2)gl)*, *rhea/talin*, *flightless (fli)*, *Tec29*, *Diaphanous (dia)*, *myospheroid (mys)*) showed a specific phenotype in either dextral (Fig 1E), sinistral (Fig 1F) or both pathways. Among the positive hits, *fli*, *chic*, *mys*, *rhea* and *DAAM* showed the strongest phenotype. In particular, loss of DAAM activity led to a strong and fully penetrant no-rotation phenotype in a *myo1D-RNAi* context. Hence, DAAM flies loose asymmetry and become symmetrical, indicating that this gene plays an essential role in sinistral development. Here, we will focus on the role of the actin nucleator DAAM in laterality.

### The formin DAAM plays a specific role in (sinistral and dextral) LR asymmetry

**Fig 2:**
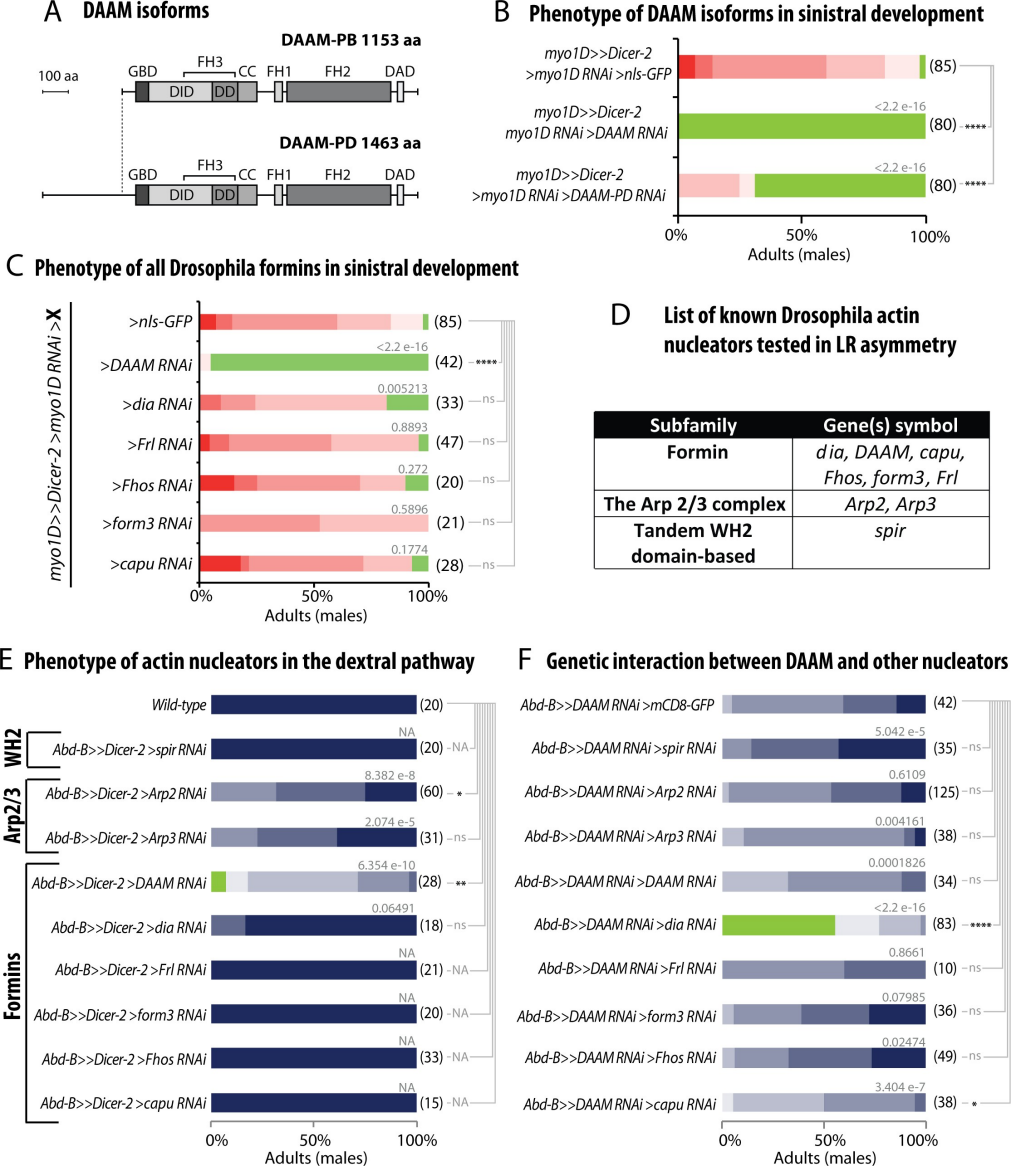
The DAAM formin plays a specific role in (sinistral and dextral) LR asymmetry. **A**, Schematic representation of the two DAAM isoforms (DAAM-PB, 1153 aa; DAAM-PD, 1463 aa) with conserved domain organization. As compared to the short isoform, the DAAM-PD long isoform contains 310 extra amino acids on the N-terminus which are encoded by a specific exon which contains no conserved protein motif. FH, Formin Homology domain; GBD, GTPase Binding Domain; DD, Dimerization Domain; CC, coiled-coiled domain; DAD, Diaphanous Auto-inhibitory Domain; DID, Diaphanous inhibitory Domain. **B**, Phenotype induced upon silencing of DAAM isoforms in sinistral development. To test for a potential sinistral activity, candidate genes are silenced in a *myo1D* loss-of-function background (*myo1D>>Dicer-2>>myo1D-RNAi* background). **C**, Phenotype induced upon silencing all Drosophila formins in sinistral development. To test for a potential sinistral activity, candidate genes are silenced in a *myo1D* loss-of-function background (*myo1D>>Dicer-2>>myo1D-RNAi* background). **D**, List of known Drosophila actin nucleators (including 6 formin genes, Arp2/3 complex and WH2 domain nucleator) which have been tested for a potential role in LR asymmetry. **E**, Phenotype induced by loss-of-function of actin nucleators in the dextral pathway. **F**, Genetic interaction between DAAM and other actin nucleators. To test genetic interaction, flies are silenced for both *DAAM* and candidate actin nucleators. In B-F, the distribution of phenotypes and statistical analysis is as described in Fig 1 legend.

**Fig 3:**
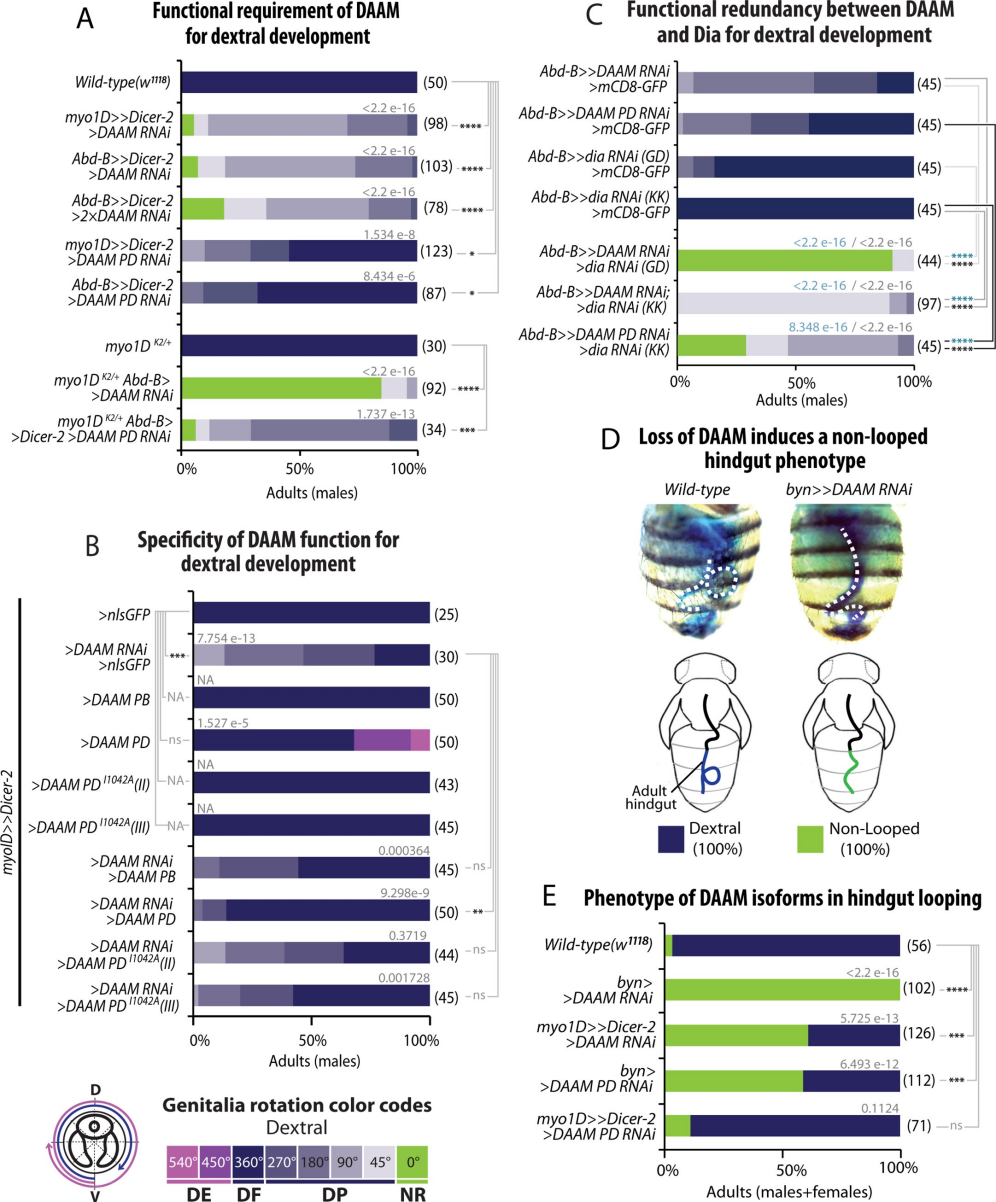
DAAM function in genitalia and hindgut LR asymmetry. **A**, Functional requirement of *DAAM* for dextral genitalia development. *DAAM* function is reduced using RNAi lines targeting both DAAM isoforms or only the long one (DAAM-PD). Genetic interaction with *myo1D* is tested in heterozygous conditions (lines 7–9). **B**, Rescue experiments showing the specificity of *DAAM* function in dextral genitalia development. While a wild type DAAM-PB or DAAM-PD can rescue the loss of *DAAM* function, a mutant form of DAAM-PD in the FH2 nucleation domain (DAAM-PD-I1042A) cannot. An over-rotation phenotype is observed upon DAAM-PD expression (this phenotype has been added to our color code: DE, extra dextral; pink arrow on genitalia scheme). **C**, The functional redundancy between *DAAM* and *dia* for dextral genitalia development has been tested by combining loss of *DAAM* and *dia* function (lanes 1–4, controls; lanes 5–7, double loss-of-function). **D**, Loss of *DAAM* function induces a non-rotated hindgut phenotype. Photographs are dorsal views of adult fly abdomens after feeding with a blue dye to reveal hindgut shape (dotted line). Phenotype schematized below photograph. **E**, Phenotype induced by loss-of-function of *DAAM* isoforms in hindgut looping. Color code for phenotype description is as described in D. In A-C and E, the distribution of phenotypes and statistical analysis is as described in Fig 1 legend.

The Drosophila *DAAM* gene encodes for two protein isoforms: a short isoform (DAAM-PB, 1153 aa) and a long one (DAAM-PD, 1463 aa) harboring an extra featureless domain (310 aa) at its N-terminus (Fig 2A)[27]. In order to test for an isoform-specific role in LR asymmetry, we compared the phenotype induced by RNAi either targeting the two isoforms (*DAAM-RNAi*) or selectively targeting the N-terminal domain of DAAM-PD (*DAAM-PD-RNAi*). Interestingly, results show that knock-down of DAAM-PD recapitulates the sinistral-to-no-rotation phenotype, suggesting a specific or primary role of the long isoform in genitalia rotation (Fig 2B). The weaker effect using this DAAM-PD RNAi line may be due to reduced level of expression or efficiency. A specific role of DAAM-PD was further confirmed by rescue experiments using expression of specific isoforms (Fig 3B). Interestingly, a form of DAAM-PD which is mutated in the FH2 nucleation domain (DAAM-PD-I1042A) could not rescue the RNAi phenotype (Fig 3B), indicating that actin assembly is essential for DAAM function in LR asymmetry.

In addition to *DAAM*, the fly genome encodes 5 other formin genes: *dia*, *Formin-related (Frl)*, *Formin homology 2 domain containing (Fhos)*, *Formin3 (form3)* and *cappuccino* (*capu*). In order to assess formin specificity in LR asymmetry, we tested the role of all formin genes in dextral and sinistral pathways. Data show that only *dia* silencing can induce a weak phenotype in the sinistral pathway (Fig 2C and Fig 1E). In addition to formins, actin can be nucleated through the tandem WH2 domain-based Spire protein and the Arp2/3 complex (Fig 2D). We thus tested the potential role of these actin nucleators in LR asymmetry. Our results show that except for a weak effect of Arp2, none of these factors showed a significant LR phenotype in either dextral (Fig 2E) or sinistral (Fig 2C) pathway. Additionally, except for *dia* showing a strong interaction (see below) and a weak effect of *capu*, their combined silencing together with DAAM-RNAi did not enhance the *DAAM* phenotype (Fig 2F). Altogether, these results suggest that the formin DAAM plays a major and specific role in LR asymmetry, likely through promoting the assembly of a specific F-actin structure important for chirality.

### DAAM is partly redundant with Diaphanous for dextral genitalia LR asymmetry

In a *myo1D-RNAi* context, the loss of DAAM induces a no-rotation phenotype, indicating an essential role in sinistral development. To test if DAAM is a general factor for LR asymmetry, we next analyzed the potential role of DAAM in dextral development. Silencing of *DAAM* by using moderate to strong drivers (*myo1D-Gal4*, *AbdB-Gal4*) led to a partial dextral phenotype, which could be increased upon doubling RNAi-expressing transgenes, leading to the appearance of some non-rotated flies (Fig 3A). Interestingly, expressing RNAi against the DAAM-PD long isoform could also induce rotation defects, confirming a role of this isoform in LR asymmetry. Of note, the *DAAM* phenotypes could almost be completely rescued upon expression of DAAM-PD (long) but less efficiently with DAAM-PB (short), further suggesting a specific role of the long isoform in LR asymmetry (Fig 3B).

The fact that i) DAAM silencing led to only partially rotated genitalia and ii) *dia* showed a weak phenotype (Fig 1E), led us to test for potential redundancy of DAAM with other formins in dextral development. We thus silenced both *DAAM* and other formins in the genitalia (Fig 2F) and found that removing both *DAAM* and *dia* significantly enhanced the *DAAM* phenotype in the dextral pathway (Fig 2F and Fig 3C). These results suggest that while *DAAM* plays an essential role in the sinistral pathway, it has a partly redundant function with *dia* for dextral development. Hence, in genitalia, the dextral pathway appears more robust than the sinistral one with regard to formin function.

To further check if the effect of DAAM is dependent on the Myo1D dextral determinant, we expressed *DAAM* RNAi in *myo1D* null heterozygous flies (*myo1D*^*k2*^*/+*). In this condition, we observed a strong enhancement of the phenotype, with 90% of the flies displaying a no-rotation phenotype. Enhancement was also induced upon knock-down of the DAAM-PD long isoform (Fig 3A). Together, these results indicate that DAAM plays a *myo1D*-dependent role in the genitalia dextral pathway, redundantly with the Dia formin.

### DAAM is a general factor controlling asymmetry of LR organs

Our results indicate a clear role of DAAM in genitalia rotation, with variable strength in the dextral and sinistral pathways. We next checked whether DAAM could also control LR asymmetry of other lateralized organs. During pupal development, the hindgut undergoes coiling which leads to the formation of a single dextral loop in adults (Fig 3D). Recent work has identified the H1 domain of the imaginal ring as a specific LR organizer for hindgut looping, in which Myo1D is specifically expressed and necessary [9]. Interestingly, we found that expression of *DAAM-RNAi* in the H1 hindgut organizer (using *bynGal4* driver) can lead to a fully penetrant non-looped phenotype (Fig 3E). As for the genitalia, expression of a *DAAM-PD*-specific RNAi can recapitulate the gut phenotype of *DAAM*, although the penetrance is not as strong. We could not test a specific role of *DAAM* in hindgut sinistral development as its silencing already leads to non-rotated hindguts in 100% of the flies. Therefore, and in contrast to genitalia, silencing of *DAAM* in the hindgut is sufficient to completely block dextral looping. Hence, DAAM can be essential (hindgut) or have partly redundant function (genitalia) with another formin for LR asymmetry. Nonetheless, these results indicate that DAAM is a general regulator of LR asymmetry in Drosophila, likely affecting all lateralized organs.

### Myo1D and DAAM are part of the same protein complex in LR organizers

**Fig 4:**
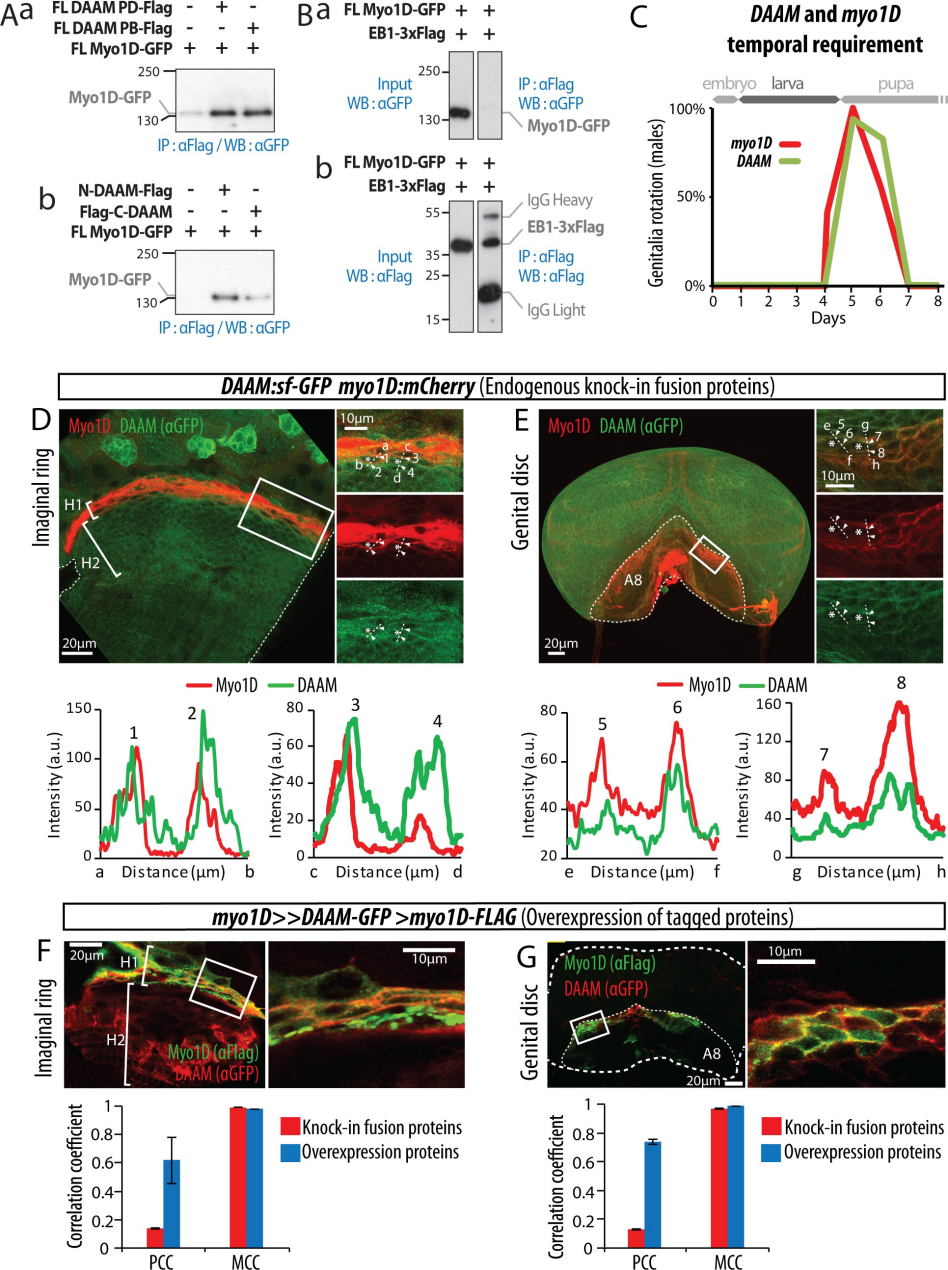
Spatial and temporal coupling of DAAM and Myo1D in LR organizer cells. **A,** Co-Immunoprecipitation experiments showing interaction between Myo1D and DAAM full length (**Aa**), N-DAAM or C-DAAM (**Ab**) in the same protein complex. **B**, Control Co-IP experiment showing that Myo1D-GFP does not show unspecific interaction with another Flag-tagged protein (EB1-3xFlag). Scale unit for blots is kDa. IgG High, IgG High Chain; IgG Light, IgG Light chain. **C**, Temporal requirement for *DAAM* and *myo1D* during genitalia rotation. Time (bottom) and developmental (upper part) scales are shown. **D**-G Intracellular localization of DAAM and Myo1D in the hindgut LR organizer cells (H1, white bracket)(**D,F**), or genital disc LR organizer cells (A8, dotted line)(**E,G**) at the third instar larval stage. Knock-in fusion proteins DAAM::GFP and Myo1D::mCherry (**D**,**E**) are expressed under the control of their respective endogenous regulatory sequences. Tagged proteins DAAM::GFP and Myo1D::Flag (**F**, **G**) are expressed under the control of the *myo1D-Gal4* driver. Images on the right are cropped single z-plane images corresponding to the white rectangles on the left panel. Plots represent fluorescence intensity profile along the dotted lines in cropped area. Quantification of DAMM and Myo1D co-localization in hindgut (**D**,**F**) and genitalia (**E,G**), using Pearson Correlation Coefficient (PCC) and Maners Co-localization Coefficient (MCC).

Myo1D has previously been shown to directly bind to DE-cadherin and β-catenin, suggesting that the adherens junction may serve as a docking platform important for Myo1D chiral activity [4,6]. Because *DAAM* and *myo1D* genetically interact, we next assessed a potential physical interaction between the two proteins, by performing co-immunoprecipitation (Co-IP) experiments from S2 cell extracts expressing tagged proteins (Fig 4A and 4B). As a control, Myo1D-GFP could not be immunoprecipitated using an EB1-3XFlag protein (Fig 4Ba,b). Full-length Myo1D tagged with GFP in combination with either full-length DAAM-PD or DAAM–PB tagged with Flag were co-expressed in S2 cells. Anti-Flag immunoprecipitation revealed that GFP-tagged Myo1D co-purified with both isoforms of DAAM (Fig 4Aa). In order to identify the Myo1D-binding domains of DAAM further, GFP-tagged Myo1D was co-expressed with either N-DAAM (aa1-430 of DAAM-PB, corresponding to aa311-aa741 of DAAM-PD; see Fig 2A) or C-DAAM (aa568-1153 of DAAM-PB, corresponding to aa878-aa1463 of DAAM-PD) both tagged with Flag. Although Myo1D co-purified with both N-DAAM and C-DAAM, the GFP signal was stronger in case of N-DAAM immunoprecipitation (Fig 4Ab), suggesting a preferential binding surface between Myo1D and the common N-terminal part of both DAAM isoforms. DAAM and Myo1D being found in the same protein complex, we next characterized their intracellular localization in LR organs. To this goal, we performed quantitative co-localization analysis of knock-in fusion proteins (Fig 4D and 4E; S1 Fig) and overexpressed proteins (Fig 4F and 4G) in genitalia and hindgut LR organizers (named A8 and H1, respectively). DAAM is found at the membrane and in puncta in both tissues. Results show that DAAM and Myo1D co-localize with cell membranes in these two cell types (Fig 4D–4G) and with DE-cadherin in hindgut LR organizer (S1 Fig), reinforcing a model of DAAM and Myo1D closely interacting in cells controlling organ asymmetry.

To further confirm this view, we determined the temporal requirement for *myo1D* and *DAAM* using the TARGET method [28], which allows the control of gene expression in space and time through a thermosensitive Gal4 driver. Results show that both genes are required during the same time window preceding genitalia rotation, between days 5–6 of development (Fig 4C)[4].

Altogether, these results support a model of DAAM and Myo1D interaction in the same protein complex in LR organizer cells, important for proper asymmetry of target organs.

### Abd-B controls DAAM expression in the genitalia LR organizer

**Fig 5:**
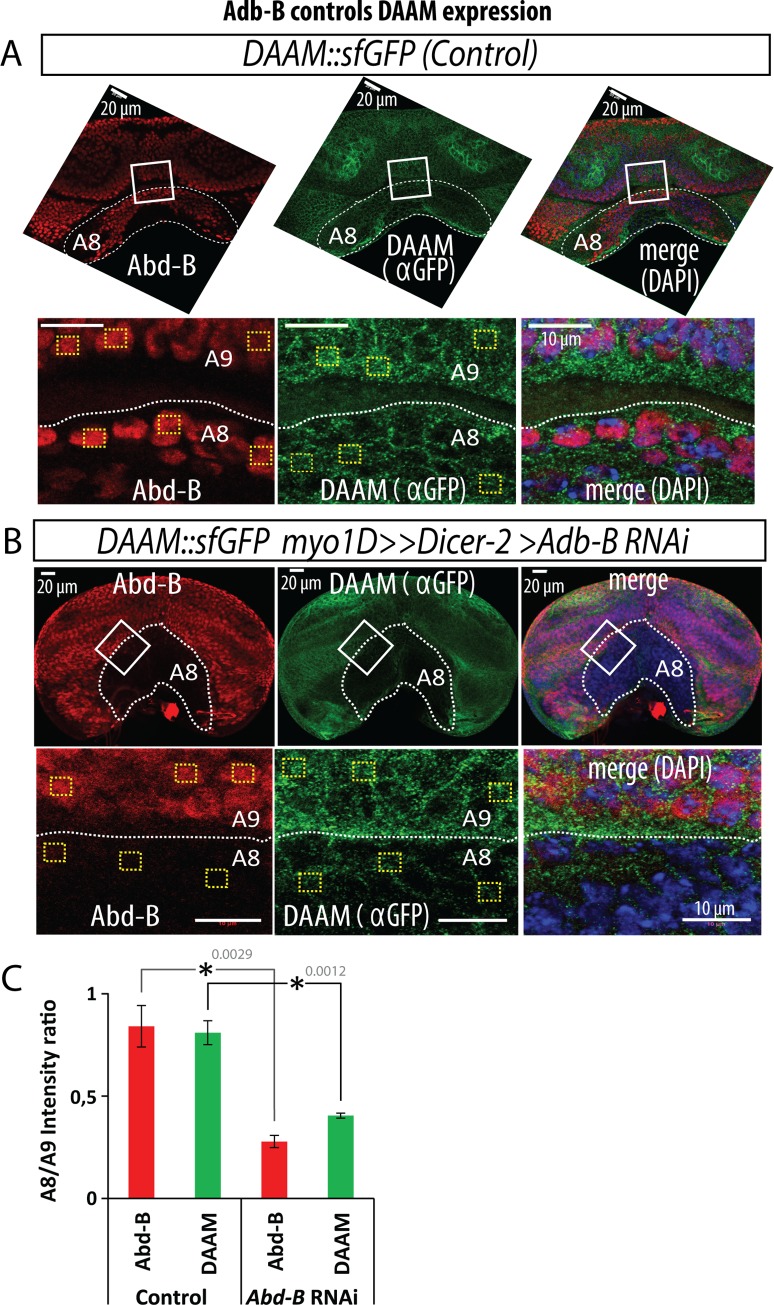
Abd-B controls the expression of DAAM in the genitalia LR organizer. **A**, Upper panel, overview of the expression of Abd-B (red) and DAAM (green) in control genitalia imaginal disc and genitalia LR organizer (A8, dotted line). Lower panel are single z-plane images at higher magnification corresponding to the region highlighted by white rectangles on the upper panel. **B**, Upper panel, overview of the expression of Abd-B (red) and DAAM (green) in genitalia imaginal disc expressing *Abd-B* RNAi against *Abd-B* in the LR organizer (A8, dotted line). Lower panel, high magnification of the expression of Abd-B and DAAM. Region corresponds to the box (white rectangle) shown in upper panel. **C**, Quantification of the reduction of DAAM and Abd-B immunostaining signal in control and *Abd-B* RNAi condition. Quantified regions correspond to yellow dotted line squares in **A** and **B**. A9 is a domain adjacent to A8 in which the *Abd-B* RNAi is not expressed. Signal intensity is calculated as an “A8 cell signal/A9 average cell signal” ratio and significance for difference between conditions assessed with a T-test. p-value is indicated in the brackets showing compared conditions. Threshold for significance of the difference between compared genotypes is defined as: *: <0.05; **: <0.01; ***: <0.001.

Previous work has shown that the expression of Myo1D in the genitalia LR organizer (A8 segment) is under the control of the Hox gene *Abd-B* for dextral development. Additionally, *Abd-B* is known to be essential for sinistral chirality as a double loss of *Abd-B* and *myo1D* leads to non-rotated genitalia [8]. Because of the dual role of DAAM in both dextral and sinistral pathways, we tested a potential regulation of the *DAAM* gene by *Abd-B*. Results show that silencing *Abd-B* specifically in the *myo1D* domain strongly reduced the expression of DAAM in the genitalia LR organizer (Fig 5A and 5B). Although we do not show direct regulation, these data suggest DAAM as a novel and major target gene of Abd-B in genitalia, essential for sinistral LR asymmetry. Therefore, in accordance with the Hox gene phenotype, Abd-B appears to control two essential factors in the genitalia LR organizer, DAAM and Myo1D, which are essential for sinistral and dextral development, respectively.

### Profilin and Tec29 cooperate with DAAM for LR asymmetry

**Fig 6:**
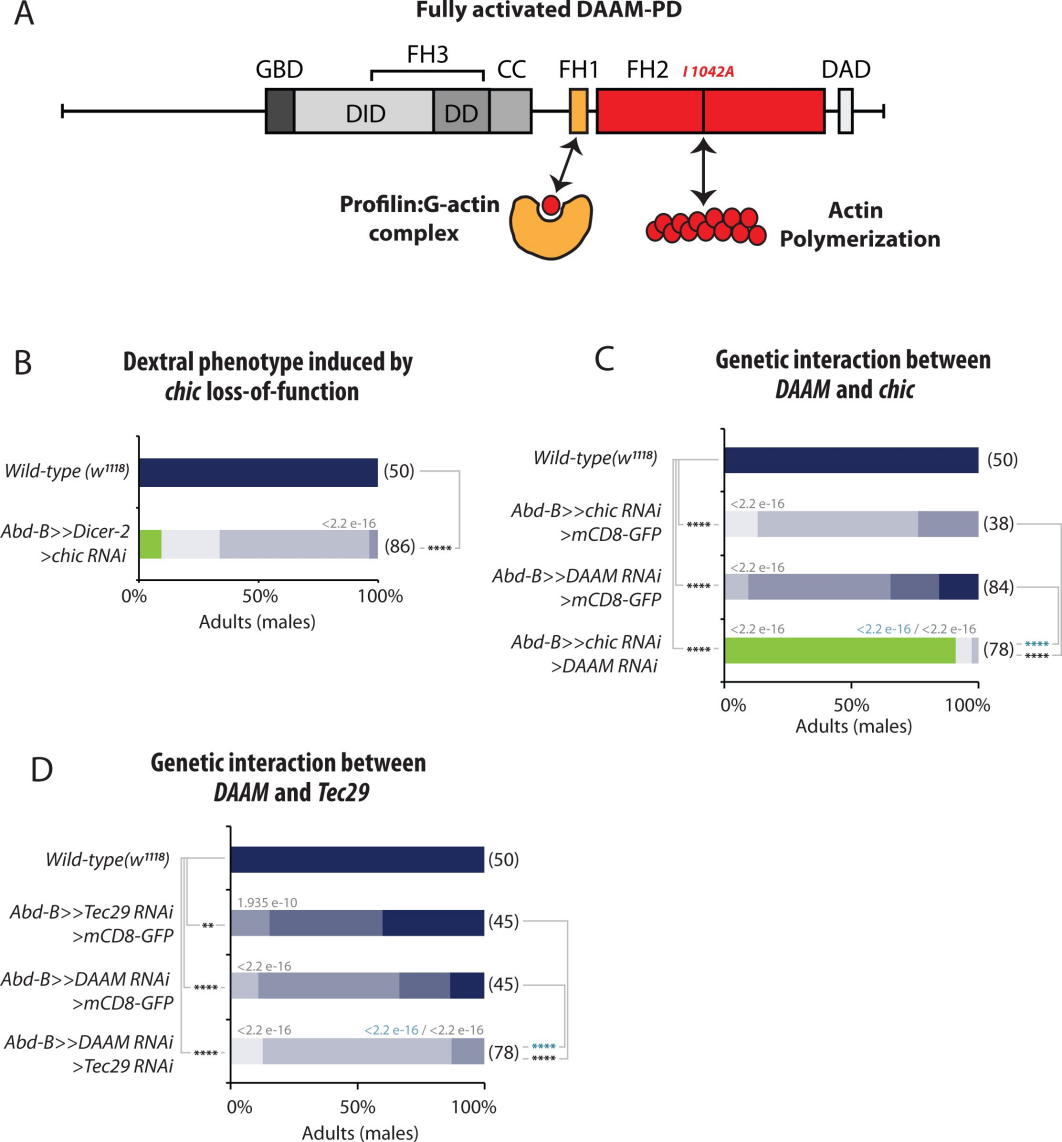
Profilin (Chic)and the Tec29 cooperate with DAAM for LR asymmetry. **A**, Schematic representation of DAAM protein domains and their role in actin dynamics. FH, Formin Homology domain; GBD, GTPase Binding Domain; DD, Dimerization Domain; CC, coiled-coiled domain; DID, Diaphanous Inhibitory Domain; DAD, Diaphanous Auto-inhibitory Domain. Profilin (Chic) binds to the FH1 domain while the FH2 domain is implicated in actin nucleation. DAAM-PD proteins bearing a point mutation in the FH2 domain (I1042A) are actin-polymerization incompetent mutant forms. **B**, Dextral phenotype induced by profilin/*chic* loss-of-function. Silencing of *chic* induces genitalia rotation defects. **C**, Genetic interaction between *DAAM* and *chic*. While silencing *chic* or *DAAM* individually leads to a weak rotation phenotype, silencing both *chic* and *DAAM* induces a strong no-rotation phenotype. **D**, Genetic interaction between *DAAM* and *Tec29*. Silencing both *DAAM* and *Tec29* significantly enhances the phenotype of either *DAAM* or *Tec29* alone. In **B-D**: Numbers in parenthesis on the right side of color bars indicate the number of individuals analyzed for each genotype; bars with different colors represent the percentage of occurrence of a given genitalia posture, as described in C. Numbers above bars indicate Wilcoxon rank sum test p-values. P-value threshold for significance of the difference between compared genotypes is defined as: *: <1e-5; **: <1e-8; ***: <1e-11; ****: <1e-14. NA: not applicable.

Similar to other formins, DAAM contains two formin homology domains, FH1 and FH2, allowing the protein to interact with Profilin/G-actin and nucleate F-actin, respectively (Fig 6A). Our genetic data show that the FH2 domains is essential for DAAM function in laterality (Fig 3B). It has been shown that DAAM controls the formation of actin filaments in several tissues, including muscles [27], trachea [24] and neurons [29]. DAAM has been shown to interact with a number of regulators modulating its nucleating activity and F-actin assembly. Of these, Profilin is an actin monomer binding protein, which is able to interact with the FH1 domain, and thought to provide monomer supply for polymerization [23]. Additional interactors include the Src family non-receptor tyrosine kinase *Tec29*, which was identified as an enhancer of the *DAAM*^*Ex1*^ tracheal phenotype [24], and *fli*, encoding a gelsolin family protein, that appears to regulate the actin assembly activity of both Daam1 and mDia in mouse cells [22]. Very interestingly, our genetic screen allowed to identify *chic*, *Tec29* and *fli* as important regulators of Drosophila LR asymmetry (Fig 1D–1F).

Silencing of *fli*, *chic* and *Tec29* either induced non-rotation (*fli*) or partial genitalia rotation defects (*fli*, *chic*, *Tec29*), indicating that like DAAM, they participate to both sinistral and dextral pathways (Fig 1D–1F). Furthermore, we could show that *DAAM* and *chic* interact genetically for LR asymmetry, as reducing their activity leads to a strong enhancement of the phenotype, with most of the flies (90%) showing non-rotated genitalia (Fig 6C). A similar interaction could also be observed with *Tec29* (Fig 6D). Altogether, these data suggest that a DAAM pathway involving *DAAM*, *Tec29* and *chic* (and potentially *fli*) is involved in the control of laterality in Drosophila.

### DAAM is essential for *de novo* LR asymmetry induced by Myo1D and Myo1C

**Fig 7:**
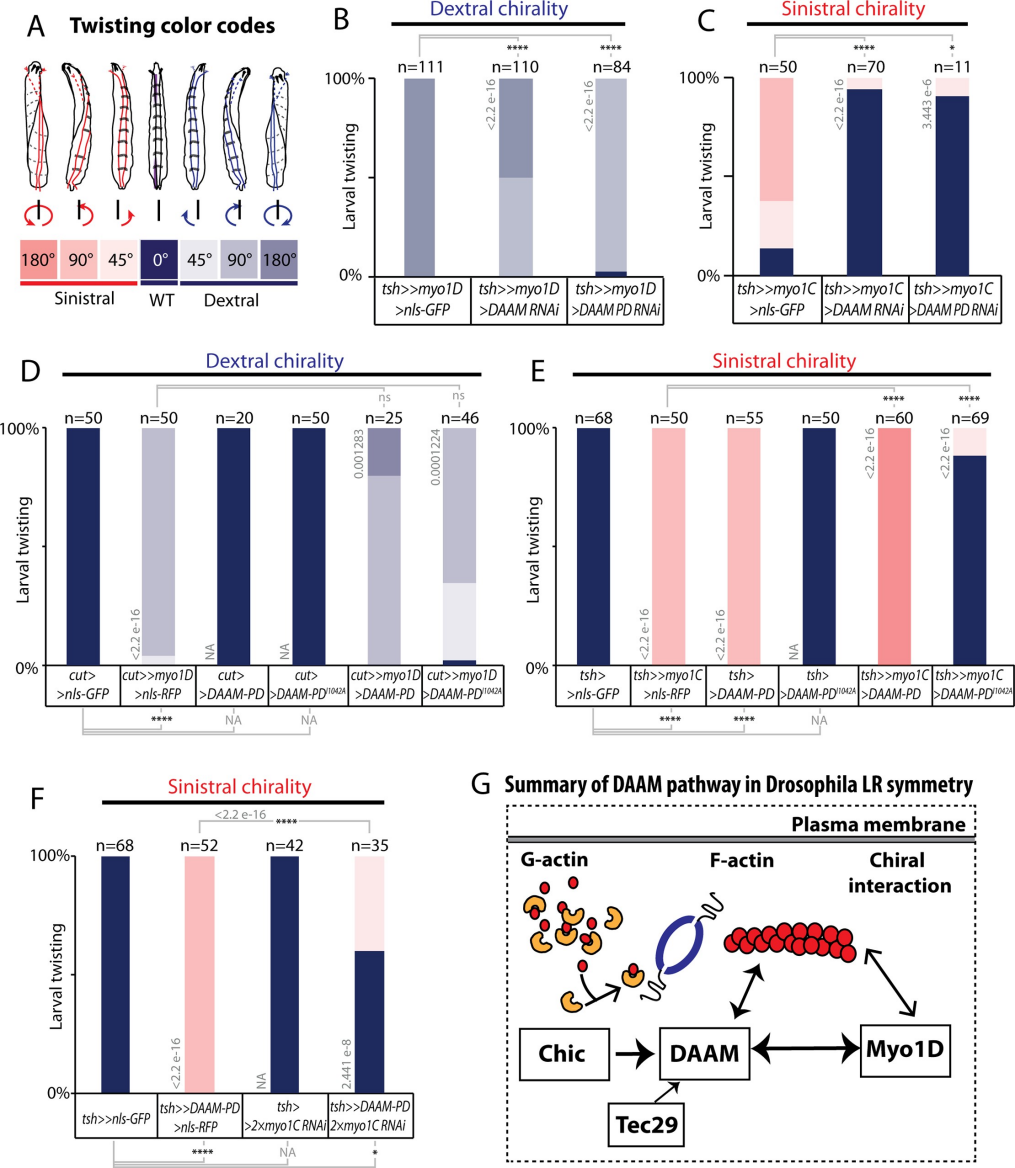
DAAM is essential for *de novo* LR asymmetry induced by Myo1D and Myo1C. **A**, Color code and schemes describing the extent of larval twisting described in B-F. Dextral and sinistral twisting is defined respectively as clockwise or anti-clockwise rotation of the anterior of the larvae when viewed from the anterior side. Anterior is up. **B**, Loss of *DAAM* function suppresses *myo1D*-induced *de novo* LR asymmetry. Removing *DAAM* function attenuates the *myo1D* phenotype.**C**, Loss of *DAAM* function suppresses *myo1C* induced *de novo* LR asymmetry. **D**, Overexpression of *DAAM* slightly enhances the *myo1D*-induced dextral twisted phenotype, while a form of DAAM that is mutant for nucleation (DAAM-PD-I1042A) does not. **E**, Overexpression of *DAAM* enhances the *myo1C*-induced sinistral twisted phenotype, while a form of DAAM that is mutant for nucleation (DAAM-PD-I1042A) does not. **F**, Loss of *myo1C* suppresses *DAAM*-induced twisting, indicating that the sinistral phenotype induced by DAAM alone is mediated through endogenous Myo1C. **G**, Summary of the Myo1D-DAAM pathway in Drosophila LR asymmetry. In **B-F**: Numbers in parenthesis on the right side of color bars indicate the number of individuals analyzed for each genotype; bars with different colors represent the percentage of occurrence of a given genitalia posture, as described in C. Numbers above bars indicate Wilcoxon rank sum test p-values. P-value threshold for significance of the difference between compared genotypes is defined as: *: <1e-5; **: <1e-8; ***: <1e-11; ****: <1e-14. NA: not applicable.

Myo1D is necessary for dextral laterality. Our recent work further showed that ectopic expression of Myo1D is sufficient to induce chirality of the whole larval body, which undergoes directional twisting (referred to as dextral by convention)[13](Fig 7B). These results indicate that Myo1D is not only necessary for native LR asymmetry but also sufficient to induce *de novo* chirality. The Drosophila genome contains a paralogous class 1 Myosin, Myosin1C, which does not play any role in LR asymmetry. However, overexpression of Myo1C can induce twisting of larval body and trachea, but with an opposite (sinistral) direction to that of Myo1D [13](Fig 7C). The chiral activity of both Myo1D and Myo1C has been shown to depend on their head motor domain and on *chic* [13]. In order to assess the role of DAAM in *de novo* LR asymmetry, we have modulated the level of *DAAM* in *myo1D* or *myo1C* gain-of-function twisted larvae. Expression of *myo1D* in the larval epidermis using the Tsh-Gal4 driver induces 180° dextral rotation of the larval body (Fig 7B). We show that co-expression of *DAAM-RNAi* suppressed this phenotype, with 50% of the larvae showing 90° dextral rotation. Interestingly, expression of a *DAAM-PD-RNAi* had a stronger suppression effect, with most larvae showing a 90° rotation and few ones with normal posture (Fig 7B). Reduction of *DAAM* could also strongly suppress the *myo1C*-induced sinistral larval twisting phenotype, indicating that DAAM is essential for *de novo* chirality induced by both Myosins (Fig 7C).

We next tested the effect of DAAM overexpression in *myo1D* or *myo1C* twisted larvae. Strikingly, co-expression of DAAM-PD with Myo1D or Myo1C led to an enhancement of the twisting phenotype induced by each myosin. While single expression of *myo1D* (using *cut-Gal4*) or *myo1C* (using *tsh-Gal4*) led to 90° dextral (Fig 7D) and 90° sinistral (Fig 7E) larvae, respectively, co-expression of DAAM-PD was able to enhance the phenotype leading to larvae twisted by 180° (Fig 7D and 7E). Of note, an FH2 domain mutant form of DAAM-PD (DAAM-PD-I1042A) could not enhance the phenotype of either myosin (Fig 7D and 7E), indicating that the actin assembly activity of DAAM is also essential for *de novo* asymmetry. These results thus indicate that DAAM is essential for *de novo* asymmetry and that it is a limiting factor for the chiral activity of Myo1D as well as that of Myo1C.

DAAM overexpression using the *cut* Gal4 line did not show any phenotype on its own (Fig 7D), while its expression using the stronger GAL4 line *tsh* led to 90° twisted sinistral larvae (Fig 7E and 7F). This result was surprising and suggested that, according to the above results, DAAM may be inducing sinistral twisting through endogenous Myo1C. To test this hypothesis, we overexpressed DAAM together with *myo1C*-RNAi (Fig 7F). In these conditions, the DAAM sinistral phenotype was strongly suppressed, further reinforcing the view that DAAM acts through Myosins to set chirality.

## Discussion

In this study, we identified the formin DAAM as an essential factor for both native and *de novo* LR asymmetry. Our results further reveal that DAAM is important not only for *myo1D*-dependent dextral chirality but also for the recessive sinistral pathway. Thus, DAAM represents a specific actin nucleator essential for all forms of Drosophila LR asymmetry.

### Actin nucleation, cell polarity and cell adhesion are important for LR asymmetry

Our genetic screen allowed us to identify a number of new regulators of LR asymmetry. Based on their known function, candidate genes can be sorted into three main categories: i) *DAAM*, *dia*, *fli*, *chic* and *Tec29*, that are likely to work together to control actin nucleation and F-actin polymerization, ii) *rhea/talin* and *mys*, which are involved in cell adhesion through integrins, and, iii) *l(2)gl* which is involved in adherens junction and apico-basal polarity. These findings are entirely consistent with current knowledge in fly and vertebrate models. First, the zebrafish *lgl2* gene has been shown to be important for E-cadherin localization at the adherens junction in the Kupffer vesicle (the LR organizer in fish), with *lgl2* mutants showing reduced vesicular lumen and cilia number [30]. Second, the Myo1D protein has been shown to directly interact with β-catenin and DE-cadherin in the genitalia [4,6], and a role of Myo1D in the chirality and remodeling of the adherens junction has been established in the embryonic hindgut [7] and adult male genitalia [31]. We therefore speculate that *l(2)gl* may be involved in the interaction between Myo1D and the adherens junction for proper LR asymmetry.

The fact that *rhea/talin* and *mys* are involved in integrin adhesion suggests an important role of cell-extracellular matrix (ECM) adhesion for genitalia LR asymmetry. Of note, mutations in the Drosophila *tenectin* gene, encoding a ligand for PS2 integrin, can induce genitalia rotation defects [32], and recent work showed that genitalia rotation involves cell intercalation and asymmetry of junction remodeling [31]. A role for cell adhesion and ECM has also been shown in vertebrates for LR asymmetry. In particular, work in chick showed that the asymmetrically expressed N-Cadherin is important for Pitx2 expression and heart looping [33]. Additionally, N-cadherin controls the asymmetry of the ECM in the dorsal mesentery that is essential for proper gut looping [34]. Future work will help to characterize the precise role played by the adhesion genes identified in our screen during Drosophila LR asymmetry and chiral morphogenesis.

While genetic screening allowed identifying new factors important for LR asymmetry, it also provided information about factors, gene families or cellular functions that are likely not being involved in the process. In particular, genes involved in microtubule formation or regulation have not been identified as potential regulators of Drosophila chirality. This observation suggests that unlike vertebrates, in which microtubules and associated molecular motors play an important role for cilia-driven flow and asymmetry, Drosophila does not depend on microtubule cytoskeleton for establishing LR asymmetry. Hence, while Drosophila rely exclusively on actin-based processes, vertebrates use both systems (actin and microtubules) for establishing LR asymmetry. Nevertheless, Myo1D is the sole common denominator between invertebrates and vertebrates [14,15], and it will be interesting to characterize how Myo1D, actin and microtubules interact for establishing vertebrate LR asymmetry.

### The DAAM pathway is essential for Drosophila LR asymmetry

Our screen and analysis show that the formin DAAM is essential for both sinistral and dextral development. Genetic invalidation of all Drosophila actin nucleators (*formins*, *spire*, *Arp2/3*) individually or in combination with DAAM shows that the formin DAAM is an LR-specific actin nucleating factor (Fig 2). Our genetic data also suggest that the long form of DAAM (DAAM-PD) may have a specific function during LR asymmetry, which might be linked to the extra N-terminal domain present in this protein isoform. Finally, we show that the FH2 actin assembly domain of DAAM is essential for both normal and *de novo* laterality (Fig 3).

Based on our results, we propose a model in which DAAM and associated regulators (Chic, FliI, Tec29) build a specific F-actin network that serves as a substrate for Myo1D function. Because Myo1D has been shown to induce chiral movement of F-actin in an *in vitro* gliding assays, we speculate that interaction between Myo1D and the DAAM-dependent F-actin network (daamF-actin) induces a chiral cytoskeleton. Since Myo1D has been shown to bind directly to DE-cadherin and β-catenin, Myo1D could serve as a scaffold and determinant at the adherens junction for assembling a DAAM-complex and a chiral cytoskeleton (Fig 7G). As a result of the formation of the tripartite complex (Myo1D, daamF-actin, AJs), cell-cell adhesion would be biased and lead to multiscale chirality of tissues and the whole body as observed in twisted larvae, trachea or genitalia [13].

Could DAAM and/or formins play a conserved role in LR asymmetry across phyla? It is interesting to note that in chick, Daam2 has been shown to be downstream of Pitx2 and Wnt for cadherin-based adhesion and cell remodeling during gut looping [35]. It would therefore be interesting to know if, in addition to Xenopus and zebrafish[14,15], Myo1D plays any role in chick LR asymmetry. Our recent work showed a link between Myo1D and Planar Cell Polarity (PCP) in Drosophila, zebrafish and Xenopus [9,15,36], and Daam1 function has been shown to be involved in PCP-mediated renal tubulogenesis [37], suggesting a conserved Myo1D-DAAM-PCP link. In the pond snail *Lymnaea stagnalis*, some natural variants show a sinistral phenotype in their shell coiling. Recent genetic linkage and mutant analysis has provided data showing that the Lymnae *diaphanous1* (*Lsdia1*) gene is important for dextral coiling of the snail shell [17–19]. These data indicate that snail depend on formin activity for their LR asymmetry. We have recently shown that chiral interaction between Myo1D and F-actin induces chirality at all biological scales [13], indicating a molecular origin of macroscopic chirality. Interestingly, work using cell culture has revealed that isolated cells can develop intrinsic chirality, leading to the self-organization and polarization of F-actin bundles. In particular, recent work showed that this process of autonomous chirality could be abolished by blocking formins, in particular *mDia1* [38,39]. In conclusion, DAAM and Dia may represent genuine formins involved in LR asymmetry in both vertebrates and invertebrates. The identification of DAAM as a LR-specific actin nucleator *in vivo* provides strong evidence that a specific subset of F-actin is assembled in LR organizers for Myo1D activity. Because DAAM is also required for *de novo* asymmetry, we conclude that DAAM is a general molecular effector helping to assemble a specific F-actin substrate for Myo1D activity in both native and *de novo* chirality.

## Materials & methods

### Drosophila strains and genetics

Details of all genotypes and transgenes can be found in FlyBase (http://flybase.bio.indiana.edu) or in references herein. The list of fly lines used in each figure is provided as a S2 Table. For the genetic screen, all crosses were made at 29°C and F1 males with appropriate genotypes were collected (N = >20) and scored for the extent of genitalia rotation (see color code, Fig 1). RNAi lines were from the Vienna Drosophila RNAi Centre (VDRC, Vienna, Austria), the NIG-Fly Stock Centre (Kyoto, Japan), and the Transgenic RNAi Project (TRiP, Boston, USA, DRSC). RNAi lines targeting the cytoskeleton were selected based on the GLAD database [25] and [26].

The following Drosophila strains have been used: *myo1D*^NP1548^-Gal4 (Kyoto #112690)[4]; *Abd-B*^LDN^-Gal4/TM3 (gift from E. Sanchez Herrero) [40]; *byn*-Gal4 (gift from K. Matsuno)[41]; *tsh*^md621^-Gal4 (Bloomington #3040)[13]; *cut*^B^-Gal4 (II) (gift from J. Casanova)[42]; *myo1D*^K2^ [4]; *DAAM*::*sfGFP* (gift from F. Schweisguth) [43]; UAS-*myo1D*::*GFP* [4]; UAS-*myo1C*::*RFP* [6]; UAS-*FL DAAM PB* [29]; UAS-*FL DAAM PD* [27]; UAS-*FL DAAM PD*^I1042A^ [27]; UAS-*myo1D* RNAi [4]; UAS-*myo1D* RNAi x2 [4]; UAS-*myo1C* RNAi x2 [6]; UAS-*DAAM* RNAi T129M [27]; UAS-*DAAM* RNAi (VDRC #103921 (KK) and VDRC #24885 (GD)); UAS-*Abd-B* RNAi (VDRC #12024 (GD)); UAS-*Arp2* RNAi (VDRC #29944 (GD)); UAS-*Arp3* RNAi (VDRC #108951 (KK)); UAS-*capu* RNAi (VDRC #34278 (GD) and VDRC #110404 (KK)); UAS-*chic* RNAi (VDRC #102759 (KK)); UAS-*dia* RNAi (VDRC #20518 (GD) and VDRC #103914 (KK)); UAS-*Fhos* RNAi (VDRC #34034 (GD)); UAS-*fli* RNAi (Bloomington #27566 (TRIP) and VDRC #39528 (GD)); UAS-*form3* RNAi (VDRC #42302 (GD) and VDRC #45594 (GD)); UAS-*Frl* RNAi (VDRC #34412 (GD)); UAS-*l(2)gl* RNAi (VDRC #51247 (GD)); UAS-*mys* RNAi (VDRC #103704 (KK)); UAS-rhea RNAi (VDRC #40400 (GD)); UAS-*spir* RNAi (Bloomington #61283 (TRIP)); UAS-*Tec29* RNAi (VDRC #106962 (KK) and Bloomington #25791 (TRIP)). The following lines were obtained from Bloomington stock center: w1118, UAS-*mCD8*::*GFP*, UAS-*nls*::*GFP*, UAS-*myr*::*RFP*, UAS-*mCD8*::*mCherry*, UAS-*nls*::*RFP*, 10xUAS-IVS-*myr*::*tdTomato*, UAS-*Dicer-2*, and *tub*-GAL80ts.

### Transgenic constructs

*myo1D*::*mNeonGreen* and *myo1D*::*mCherry*: These line are generated by CRISPR/Cas9 mediated in-frame insertion of a “GVG-linker-mCherry” or “GVG-linker-mNeonGreen” coding sequence on the C-terminal side of *myo1D* coding sequence (InDroso service). UAS-*myo1D*::*Flag*: the cDNAs for full length Drosophila Myo1D, encoding amino acids 1–1011, were amplified by PCR with primers that added a NotI site at the 5’ end and “Avi-FLAG” tag and KpnI site at the 3’ end. The PCR products was inserted into pUASt vector. For each construct, several independent transgenic lines were generated (BestGene service) and tested.

The UAS-*DAAM PD*::*GFP*^4A^ line was created in a similar way as UAS-DAAM PD [27] by using the pTWV Drosophila Gateway vector. The DAAM PD isoform specific RNAi line (UAS-DAAM PD RNAi) was created by cloning a PD region specific sequence (AATGAGTGTCCTCATGGATAA) into pValium20 that was subsequently inserted into the attP2 landing site.

### Temporal requirement assay

Double temperature-shift experiments to determine the temporal window for *myo1D* and *DAAM* function in genitalia rotation were performed using the TARGET method [28]. In brief, flies with *TUB-GAL80*^*ts*^, *AbdB-Gal4*, *UAS-Dicer2*,*UAS-X-RNAi* were synchronized at 25°C to collect embryos of different developmental stages (day 1 to day 8). Collected embryos were shifted to 30°C for 1 day (RNAi expression). Data were collected at each time point (day1- 8, N = 30) and plotted.

### Dissection, immunohistochemistry and image acquisition

Dissection of genital discs and larval imaginal rings was done as previously described [6][9]. Antibodies used are primary: goat anti-GFP (Antibodies-on-line, 1:400), mouse anti-AbdB (DSHB, 1:400), rabbit anti-DE-cadherin (DSHB, 1:50), mouse anti-mNeon:Green (32F6—Chromotek, 1:400) and rabbit anti-Flag (Sigma, 1:400), and secondary: donkey anti-goat-Alexa488 (A11055 –Life technologies, 1:500), donkey anti-goat-Alexa546 (A11056 –Life technologies, 1:500), donkey anti-mouse-Alexa488 (A21202 –Life technologies, 1:500), donkey anti-rabbit-Alexa488 (A21206-Life technologies, 1:500), donkey anti-rabbit-Alexa546 (A10040-Life technologies, 1:500) and Goat anti-rat-Alexa546 (A11081-Life technologies, 1:500). FITC-Phalloidin (P5282-Sigma, 1:500) was incubated overnight at +4°C with secondary antibodies. Samples were imaged with a Zeiss Z780 confocal microscope with the Zen software and post-treated with the Fiji Software. Co-localization studies were done using the JaCoP plugin of ImageJ. Pearson's Correlation Coefficient (PCC) and Manders co-localization coefficient (MCC) were calculated using the JaCoP plugin in endogenous and over-expressed conditions for DAAM and Myo1D proteins. For signal intensity quantifications in Figs 5, S1 and S2: Maximum signal intensity of regions of interest was determined on a single z-plane using the Fiji Software. A “Gal4-positive cell maximum signal/‘Gal4-negative cells’ average maximum signal” ratio was determined for each analyzed condition. Comparison of these ratio was used for statistical analysis.

### Co-immunoprecipitation and western blots

S2 cell transfection and sample preparation were performed as described previously [44]. S2 cell lysates were incubated with anti-Flag coated agarose-beads (Sigma) and after an intensive wash, proteins were eluted in standard Laemmli buffer. Protein samples were subjected to SDS-PAGE and analyzed by western blot. Rabbit anti-GFP (1:2000, Invitrogen), mouse anti-Flag (1:5000, Sigma), anti-rabbit IgG-HRP (1:10000, Jackson) and anti-mouse IgG-HRP (1:5000, Jackson) were used for western blots. The EB1 coding sequences were amplified from a BDGP cDNA clone (LD08743), first cloned into pENTR3C and subsequently inserted into the pAWF vector by using standard Gateway cloning methods before using for S2 cell transfections.

### Statistical analysis

Statistical analysis was conducted using RStudio v3.5.1 and Wilcoxon rank sum test for Figs 1–4, 6 and 7. Statistical analysis for Fig 5 and S2 and S3 Figs was conducted using Microsoft Excel and one-tailed Student T-test. For Figs 1–4, 6 and 7 p-value threshold for significance of the difference between compared genotypes is defined as: *: <1e-5; **: <1e-8; ***: <1e-11; ****: <1e-14. For Fig 5 and S2 and S3 Figs p-value threshold for significance of the difference between compared genotypes is defined as: *: <0.05; **: <0.01; ***: <0.001.

## Supporting information

S1 Fig (related to Fig 4). DAAM and Myo1D proteins are enriched with the cell membrane (**A**) and associated with DE Cadherin at the adherens junction (**B**) in the adult hindgut LR organizer (H1, white brackets). Cell membranes are visualized with expression of membrane targeted mCherry under the control of the *byn-Gal4* driver. Adherens junctions are visualized using a DE-cadherin (DE-Cad) antibody. Images on the right are single z-plane images at higher magnification corresponding to the region highlighted by white rectangles on the left panel. Plots represent fluorescence intensity profile along the dotted lines in high magnification images. Schemes describe position of the plot profile with respect to the imaged cell orientation. H2, hindgut precursor cells domain (white bracket).
(PDF)

S2 Fig (related to Fig 4). DAAM expression in gut and genitalia organizer is not affected upon *myo1D* depletion.
**A,** Expression of DAAM in the larval hindgut LR organizer cells (H1, white bracket), and hindgut precursors (H2, yellow bracket) domain, or larval and pupal genital discs LR organizer cells (A8) in control and *myo1D* RNAi condition. DAAM::GFP knock-in fusion proteins expressed under the control of its respective endogenous regulatory sequences is detected with anti-GFP antibody. Gut and genitalia organizer cells are visualized with the expression of tdTOMATO under the control of the *myo1D*-Gal4 driver. Images on the right–or bottom right for pupal genitalia—are cropped single z-plane images corresponding to the white rectangle region(s) on the left panel. For quantification of the DAAM immunostaining signal in control and *myo1D* RNAi condition, maximum signal intensity in regions corresponding to white and yellow lines in single z-plane images were obtained, then a signal intensity ratio is calculated as an “*myo1D*-Gal4-positive cell signal (white line)/*myo1D*-Gal4-negative cell average signal (yellow lines)”. Significance for difference between conditions is assessed with a T-test. p-value is indicated on the brackets showing compared conditions. Threshold for significance of the difference between compared genotypes is defined as: *: <0.05; **: <0.01; ***: <0.001. ns: non-significant
**B**, DAAM and Myo1D are both expressed in genitalia’s A8 domain at the pupal stage. Knock-in fusion proteins DAAM::GFP and myo1D::mCherry are expressed under the control of their respective endogenous regulatory sequences and detected using anti-GFP and anti-RFP antibodies, respectively.
(PDF)

S3 Fig (related to Fig 4). Actin expression and subcellular localization in gut and genitalia organizer is not affected upon DAAM depletion. Actin expression in the larval hindgut LR organizer cells (H1, white bracket), and hindgut precursors (H2, yellow bracket) domain, or larval discs LR organizer cells (A8) in control and *DAAM* RNAi condition. Actin is detected using FITC-conjugated Phalloidin and gut and genitalia organizer cells are visualized with the expression of RFP under the control of the *myo1D*-Gal4 driver. Images on the right are cropped single z-plane images corresponding to the white rectangle region(s) on the left panel. Single z-plane images for the gut show lateral views of the cell with on their basal side (bottom of the image) the intensely stained muscular sheet. For quantification of the Actin staining signal in control and *DAAM* RNAi condition, maximum signal intensity in regions correspond to white and yellow lines in single z-plane images were obtained, then a signal intensity ratio is calculated as an “*myo1D*-Gal4-positive cell signal (white line)/*myo1D*-Gal4-negative cell average signal (yellow lines)”. Significance for difference between conditions is assessed with a T-test. p-value is indicated on the brackets showing compared conditions. Threshold for significance of the difference between compared genotypes is defined as: *: <0.05; **: <0.01; ***: <0.001. ns: non-significant.
(PDF)

S1 Table (related to Fig 1). List of the cytoskeletal genes tested in the screen described in Fig 1B., indicating Gene ID, Gene name/Symbol, RNAi identifier, Source and phenotype.
(XLSX)

S2 Table (related to Figs 1–7, S1–S3 Figs). List of fly genotypes used in each of the figures.
(XLSX)

S3 Table (related to Figs 1–7, S1–S3 Figs). Raw counting and staining intensity data.
(XLSX)
