# Supplementary figures and images for: The Drosophila actin nucleator DAAM is essential for left-right asymmetry
Source: PLoS Genet. 2020 Apr 23;16(4):e1008758. doi: 10.1371/journal.pgen.1008758 (PMC7200016; doi:10.1371/journal.pgen.1008758)

**S1 Fig. Chougule et al.**

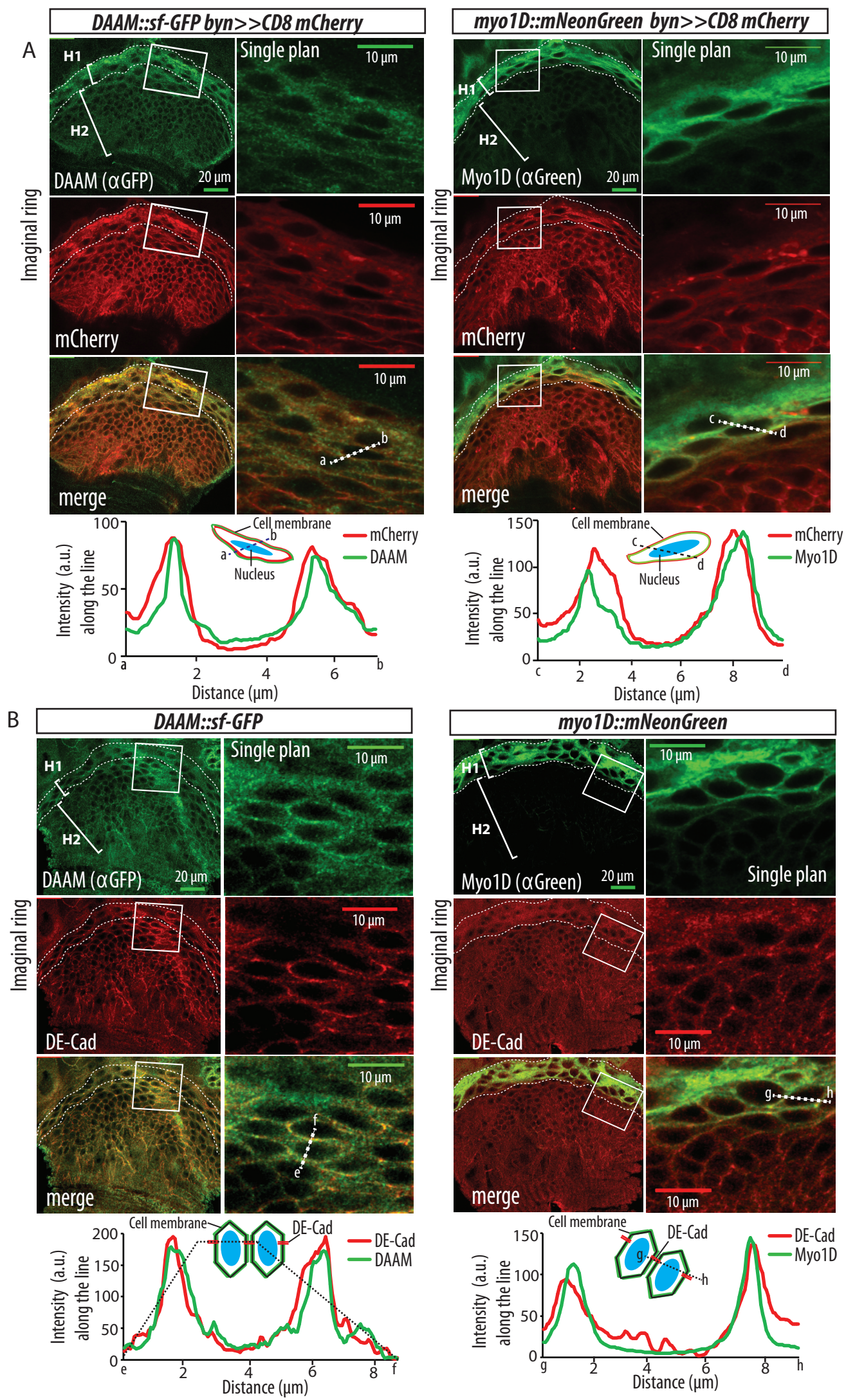

Supplement: S1 Fig — DAAM and Myo1D proteins are enriched with the cell membrane (A) and associated with DE Cadherin at the adherens junction (B) in the adult hindgut LR organizer (H1, white brackets). Cell membranes are visualized with expression of membrane targeted mCherry under the control of the byn-Gal4 driver. Adherens junctions are visualized using a DE-cadherin (DE-Cad) antibody. Images on the right are single z-plane images at higher magnification corresponding to the region highlighted by white rectangles on the left panel. Plots represent fluorescence intensity profile along the dotted lines in high magnification images. Schemes describe position of the plot profile with respect to the imaged cell orientation. H2, hindgut precursor cells domain (white bracket). (PDF) [file pgen.1008758.s001.pdf]

S2 Fig. Chougule et al.

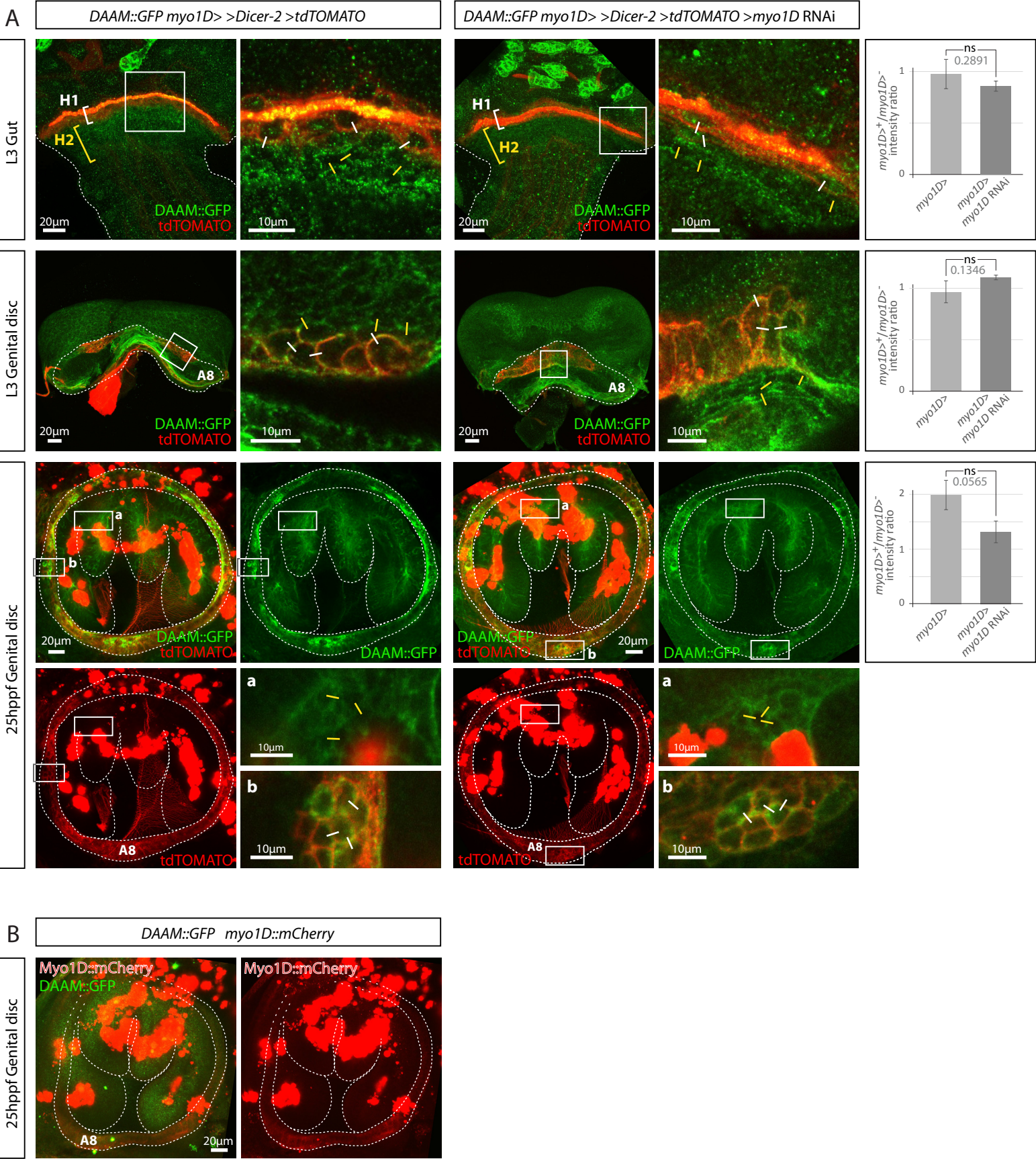

Supplement: S2 Fig — DAAM expression in gut and genitalia organizer is not affected upon myo1D depletion. A, Expression of DAAM in the larval hindgut LR organizer cells (H1, white bracket), and hindgut precursors (H2, yellow bracket) domain, or larval and pupal genital discs LR organizer cells (A8) in control and myo1D RNAi condition. DAAM::GFP knock-in fusion proteins expressed under the control of its respective endogenous regulatory sequences is detected with anti-GFP antibody. Gut and genitalia organizer cells are visualized with the expression of tdTOMATO under the control of the myo1D-Gal4 driver. Images on the right–or bottom right for pupal genitalia—are cropped single z-plane images corresponding to the white rectangle region(s) on the left panel. For quantification of the DAAM immunostaining signal in control and myo1D RNAi condition, maximum signal intensity in regions corresponding to white and yellow lines in single z-plane images were obtained, then a signal intensity ratio is calculated as an “myo1D-Gal4-positive cell signal (white line)/myo1D-Gal4-negative cell average signal (yellow lines)”. Significance for difference between conditions is assessed with a T-test. p-value is indicated on the brackets showing compared conditions. Threshold for significance of the difference between compared genotypes is defined as: *: <0.05; **: <0.01; ***: <0.001. ns: non-significant B, DAAM and Myo1D are both expressed in genitalia’s A8 domain at the pupal stage. Knock-in fusion proteins DAAM::GFP and myo1D::mCherry are expressed under the control of their respective endogenous regulatory sequences and detected using anti-GFP and anti-RFP antibodies, respectively. (PDF) [file pgen.1008758.s002.pdf]

S3 Fig. Chougule et al.

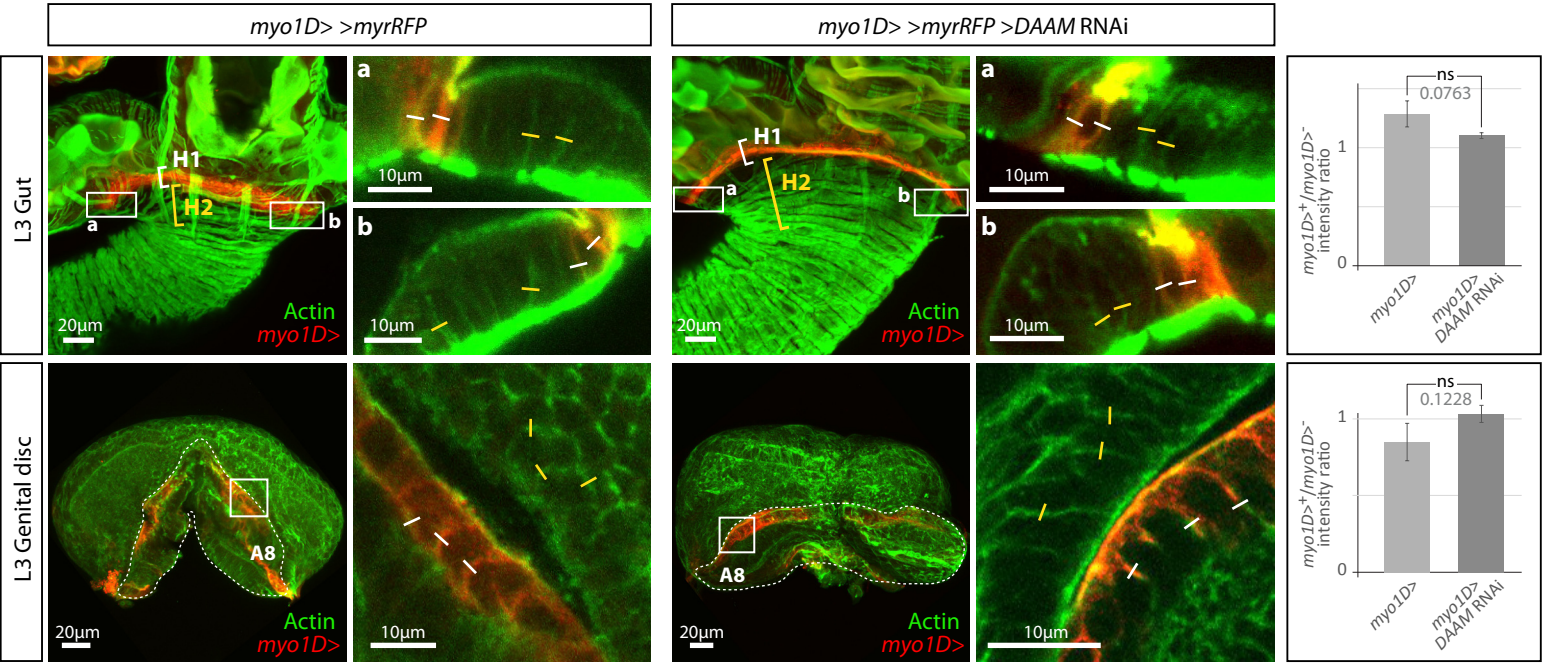

Supplement: S3 Fig — Actin expression and subcellular localization in gut and genitalia organizer is not affected upon DAAM depletion. Actin expression in the larval hindgut LR organizer cells (H1, white bracket), and hindgut precursors (H2, yellow bracket) domain, or larval discs LR organizer cells (A8) in control and DAAM RNAi condition. Actin is detected using FITC-conjugated Phalloidin and gut and genitalia organizer cells are visualized with the expression of RFP under the control of the myo1D-Gal4 driver. Images on the right are cropped single z-plane images corresponding to the white rectangle region(s) on the left panel. Single z-plane images for the gut show lateral views of the cell with on their basal side (bottom of the image) the intensely stained muscular sheet. For quantification of the Actin staining signal in control and DAAM RNAi condition, maximum signal intensity in regions correspond to white and yellow lines in single z-plane images were obtained, then a signal intensity ratio is calculated as an “myo1D-Gal4-positive cell signal (white line)/myo1D-Gal4-negative cell average signal (yellow lines)”. Significance for difference between conditions is assessed with a T-test. p-value is indicated on the brackets showing compared conditions. Threshold for significance of the difference between compared genotypes is defined as: *: <0.05; **: <0.01; ***: <0.001. ns: non-significant. (PDF) [file pgen.1008758.s003.pdf]
